# Cell type-specific intercellular gene transfer in mammalian cells via transient cell entrapment

**DOI:** 10.1038/s41421-021-00359-x

**Published:** 2022-03-01

**Authors:** Quanbin Xu, Xiaojuan Zhang, Gilson J. Sanchez, Adrian T. Ramirez, Xuedong Liu

**Affiliations:** 1 Department of Biochemistry, University of Colorado, 3415 Colorado Ave, JSCBB, 596 UCB, Boulder, CO USA; 2 Department of MCD-Biology, University of Colorado, Boulder, CO USA

**Keywords:** Breast cancer, RHO signalling

Dear Editor,

The transfer of genetic information between different cells and organisms, known as horizontal gene transfer (HGT), is one of the key mechanisms for acquiring new functions during the evolution of prokaryotic and eukaryotic genomes^1^. The mode and consequences of HGT are well established for prokaryotes and fungi^2^. Still, the evidence for cell-contact-dependent HGT between different cell types of mammalian cells that result in persistent changes remains scarce due to evolutionary barriers^2^. Here we report a pair of mammalian cell lines that engage in intercellular gene transfer in real-time at a very high frequency. We found the intercellular transfer of a fluorescent reporter gene occurs exclusively between the cell pair and requires an mRNA intermediate and reverse transcription. Robust gene transfer also requires direct cell–cell contact and is facilitated by a unique transient cell entrapment process forming an intercellular mosaic structure which requires ROCK kinase-dependent actin rearrangement in recipient cells. Our study reveals the existence of a novel mode of intercellular gene transfer in mammalian cells.

In an experiment to study the mitochondrial recruitment of Parkin, a critical regulator of mitophagy, we cocultured RPE1, an immortalized non-transformed pigmented epithelium cell line stably expressing Venus-Parkin, and MDA-MB-231, a metastatic breast cancer cell line stably expressing H2B-mCherry. Unexpectedly, after 48 h of coculture, we observed that ~20% of the mixed cell populations became double-positive for both Venus-Parkin and H2B-mCherry by both confocal microscopy and flow cytometry analysis (Fig. 1a, b). The double-positive cells were isolated by cell sorting and further analyzed by karyotype analysis. All double-positive cells carried 46 chromosomes plus a small marker chromosome (*n* = 10, Supplementary Fig. S1a), which is identical to the karyotype of the parental diploid RPE1 cells. The ratios of double-positive cells peaked at 96 h and declined at later time points, which might be a result of the higher rate of proliferation of non-transduced RPE1 cells, or reduced proliferation rate of parental MDA-MB-231 cells in the cocultured environment, or reduced cell–cell interaction when the confluence of coculture cells increases (Supplementary Fig. S1b). Double-positive cells (referred to as RPE1mut231) from coculture and cell sorting were stable as they could be perpetuated indefinitely (Supplementary Fig. S1c, d). PCR analysis of the genomic DNA isolated from RPE1mut231cells showed that the H2B-mCherry DNA was present in these cells but not in the parental RPE1 cells (Supplementary Fig. S1e), suggesting that H2B-mCherry has been stably transferred from MDA-MB-231 to RPE1. To further confirm gene transfer, we performed retroviral integration site analysis using the GenomeWalker technology with both cell lines. This analysis conclusively identified at least one MoLV-H2B-mCherry site in the donor cell on chromosome 11 within an interspersed repetitive (MIR) element sandwiched by two partial Line-1 elements. This region contains several ENCODE candidate cis-regulatory elements featuring high levels of the H3K27Ac mark (Supplementary Fig. S1f, g). In RPE1mut231 cells, one recovered site showed that the MoLV-H2B-mCherry transgene (3C2ndPCR) is integrated 3-bp upstream of the initiation codon (ATG) of ORF2 of a Line-1 element L1PA4 that resides in CYP3A51P pseudogene on chromosome 7. The integration sites for H2B-mCherry appear to be distinct in the donor and recipient cells as confirmed by sequencing and locus-specific PCR analysis (Fig. 1c and Supplementary Fig. S1f–h). Although this analysis by no means can identify all possible integration sites of the H2B-mCherry gene in both cell lines or implicate which transgene is more likely on the move, identification of nonidentical integration sites between donor and recipient cells supports the conclusion that intercellular gene transfer occurred upon coculturing these two cell lines.

**Fig. 1:**
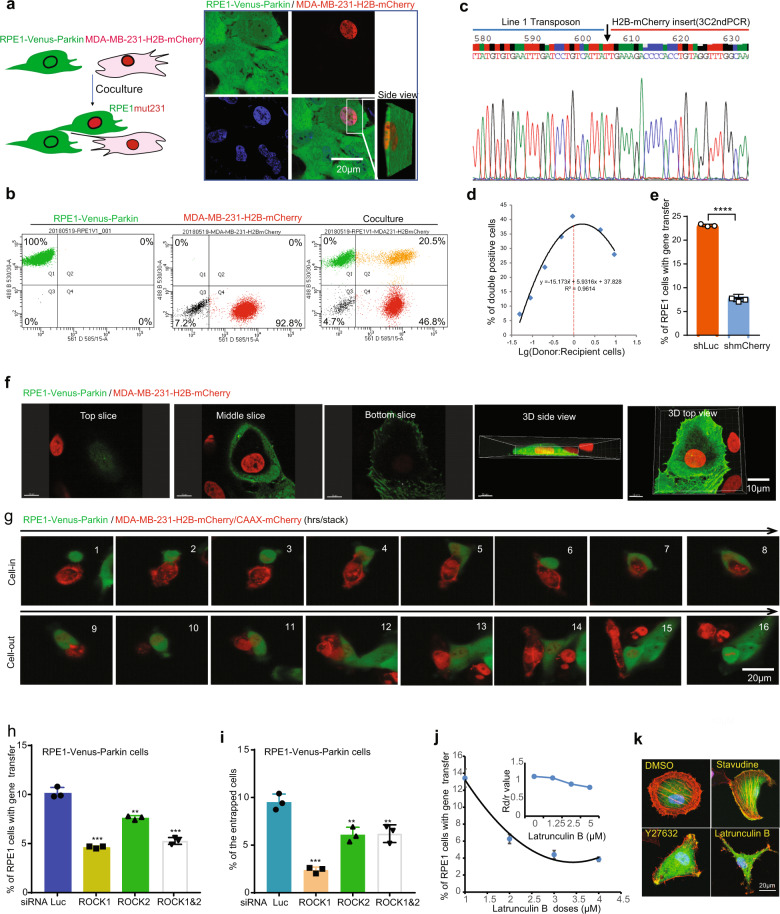
Intercellular gene transfer via cell entrapment. **a** Schematic of the coculture experiment and the confocal images of RPE1-Venus-Parkin, MDA-MB-231-H2B-mCherry, and a recipient positive for both Venus-Parkin and H2B-mCherry signals. **b** Flow cytometric analysis of RPE1-Venus-Parkin, MDA-MB-231-H2B-mCherry, and recipient cells showing both Venus-Parkin and H2B-mCherry signals. **c** An integration site of MoLV reporter transgene in the genome of recipient cell RPE1mut231. **d** Effect of the donor to recipient cell ratio (Rd/r) on gene transfer frequency. The donor to recipient cell ratio (Rd/r) was calculated as the number of donor cells (Q3 + Q4) divided by the number of recipient cells (Q1 + Q2). **e** Flow cytometric analysis of gene transfer between RPE1-Venus-Parkin and MDA-MB-231-H2B-mCherry cells stably expressing mCherry-shRNA or a luciferase gene. The mCherry-shRNA, Rd/r = 0.76 ± 0.06; shRNA control, Rd/r = 0.53 ± 0.03. **f** Confocal images of the cell-in-cell structure formed by RPE1-Venus-Parkin and MDA-MB-231-H2Bm-Cherry. **g** Confocal live-cell imaging of cell entrapment between RPE1 and MDA-MB-231-H2B-mCherry/CAAX-mCherry. The upper panel shows the engulfment of a donor cell by the recipient cell; the bottom panel shows the exit of a donor cell from the recipient cell. **h** Flow cytometric analysis of gene transfer between MDA-MB-231-H2B-mCherry and RPE1-Venus-Parkin with siRNA against luciferase, *ROCK1*, *ROCK2*, or *ROCK1* plus *ROCK2*. **i** Effect of ROCK kinase depletion on cell entrapment between MDA-MB-231-H2B-mCherry and RPE1-Venus-Parkin with siRNA against luciferase, *ROCK1*, *ROCK2*, or *ROCK1* plus *ROCK2*. **j**, **k** Effect of Latrunculin B on intercellular gene transfer and F-actin stability (Alexa Fluor™ 594 Phalloidin). Data were means ± SD; statistical significance for **e, h** and **i** was assessed using the student’s *t-*test (****P < 0.0001).

Next, we set out to optimize the coculture system for achieving the highest efficiency of gene transfer. Specifically, we investigated whether different ratios of donor and recipient cells affect gene transfer and found that the occurrence of RPE1mut231 peaked around 40% when cocultured donor and recipient cell lines mixed at a 1:1 ratio (Fig. 1d). Variability of the efficiency of gene transfer was statistically insignificant when the mixing ratio (R_d/r_) of the donor to recipient cells stays in specific ranges (i.e., 0.8 vs 0.9~0.8 vs1.9) based on post hoc analysis (Supplementary Table S1). These analyses confirm that robust gene transfer occurs from MDA-MB-231 (donor) to RPE1 (recipient) cells.

We investigated whether transcription of H2B-mCherry in donor cells is required for the transfer of this reporter gene. The knockdown of H2B-mCherry using shRNA in MDA-MB-231 significantly blocks the appearance of mCherry signals in recipient cells (Fig. 1e and Supplementary Fig. S2a–c), suggesting gene transfer requires an mRNA intermediate. Given MoLV reporter transgene is integrated into the genome of recipient cells, we postulated that reverse transcription of mRNA intermediate is indispensable for this process. In line with this, adding Stavudine, an inhibitor of reverse transcriptase, to a coculture of donor and recipient cells significantly reduced the percentage of RPE1mut231 cells (Supplementary Fig. S2d). Notably, the inhibitory effect of Stavudine on reverse transcriptase occurred only in donor cells (Supplementary Fig. S2e), suggesting that the reverse transcription of the reporter gene in donor cells, but not in recipient cells, is required for intercellular gene transfer.

We further characterized the potential mediators of this process. Neither the cultured supernatant from MDA-MB-231 (H2B-mCherry) cells nor extracellular vesicles obtained by ultracentrifugation of the donor cell supernatants (100,000 × *g*), were able to transfer H2B-mCherry to RPE1 cells (Supplementary Fig. S3a–e). Consistently, physical separation of donor and recipient cells reciprocally by the Transwell insert inhibited any detectable transfer by flow cytometry analysis (Supplementary Fig. S3f). These results are inconsistent with a cell-free transfer of genetic information via diffusible nucleoprotein complex, viruses, or extracellular vesicles^3^, suggesting that cell–cell contact is required to achieve intercellular gene transfer.

To visualize the dynamics of cell interactions and gene expression during the transfer, we performed high content live-cell imaging of the interaction between the donor and recipient cells for a period of 11 to 24 h. The most notable feature upon the coculturing of donor and recipient cells was the formation of the “cell-in-cell” like mosaic structure. The donor MDA-MB- 231 (H2B-mCherry) cells were found trapped inside the recipient RPE1(Venus-Parkin) cells frequently while maintaining their cellular boundaries (Fig. 1f, g, Supplementary Fig. S4a, c, d, and Movies S1, S2). The detention of donor cells by recipient cells was a transient and dynamic process. The percentage of cell mosaic structures increased significantly from 7 to 15 h and decreased slowly after 19 h, while the signal intensity of H2B-mCherry in the recipient cells’ nuclei steadily increased over time (Fig. 1g and Supplementary Figs. S1d, S5d). The donor and recipient cells’ engagement in the cell mosaic state was reversible as the resolution of this state often occurs within hours. As high as 20% of donor cells entered the state of entrapment during the time course of imaging (Supplementary Fig. S5d), which closely matched the efficiency of gene transfer as determined by flow cytometry analysis (Fig. 1b). The entrapment of the donor cells to form the cell mosaic state is cell type-specific since this process was not observed between RPE1(Venus-Parkin) and HeLa (H2B-mCherry) (Supplementary Fig. S4b and Movie S3). Thus, these observations suggest that cell entrapment is a cell type-specific process.

The formation of cell mosaic structure may require coordinated intercellular communications and probably specific receptor–ligand interactions. To identify the cellular effectors that regulate intercellular gene transfer, we performed a small set of mRNA knockdown screening with genes known to be involved in entosis^4^ and uncovered ROCK kinase 1/2 as strong hits for this process (Supplementary Fig. S6b). Depletion of ROCK1 and ROCK2 using siRNAs in recipient cells can abrogate both gene transfer and cell entrapment (Fig. 1h, i). In contrast, depleting ROCK1 and ROCK2 in donor cells has no obvious effects on gene transfer (Supplementary Fig. S5a, b). The effect of ROCKs perturbation by RNA depletion was phenocopied by the treatment of ROCK kinase inhibitor Y27632 (Supplementary Fig. S5c, d). The perturbation of gene transfer is unlikely due to inhibition of cell proliferation and motility as both are barely affected by ROCK1/2 inactivation in either donor or recipient cells (Supplementary Fig. S5e–h).

Since ROCK kinases are involved in actin cytoskeleton organization, we further tested whether Latrunculin B, an actin polymerization inhibitor, can block this process. As expected, the incidence of gene transfer was significantly decreased, along with F-actin levels in the presence of Latrunculin B (Fig. 1j, k). These results underscore the importance of actin dynamics as a key regulator of cell entrapment and intercellular gene transfer.

The process described here, at first glance, shares some similarities to entosis. For example, both require ROCK kinases activity. However, they are fundamentally different in several aspects. First, E-cadherin is required for entosis^5^. MDA-MB-231 cells do not express E-cadherin and are known to be incompetent to undergo entosis^6,7^; yet these cells can pair with RPE1 to perform gene transfer. Secondly, depletion of CDC42 kinase can trigger mitotic entosis in adherent cells. However, depletion of CDC42 in donor or recipient cells has the opposite effect on gene transfer: gene transfer decreased when CDC42 was depleted in recipient cells or inhibited by ML141 inhibitor in cocultured cells but increased when it was depleted only in donor cells^8^ (Supplementary Fig. S6a, b). This observation indicates that induction of mitosis in donor cells, rather than recipient cells, can promote gene transfer (Supplementary Fig. S6c). Finally, entosis is a rare event that involves whole chromosome gains or losses due to cytokinesis failure^5^. Gene transfer by cell entrapment is highly efficient without significant changes in karyotypes (Supplementary Fig. S1a). Robust intercellular gene transfer is probably an intrinsic property of MDA-MB-231 as the H2B-mCherry gene in other cell lines (i.e., HeLa) can barely be transferred to RPE1-Venus-Parkin cells (Supplementary Fig. S6d, e). A similar transfer frequency of H2B-mCherry can be achieved with independently generated MDA-MB-231 stable cells. However, α-Tubulin-mCherry in MDA-MB-231 was poorly transferred to RPE1-Venus-Parkin cells (<2%) (Supplementary Fig. S6d, e). The intercellular gene transfer between MDA-MB-231 (H2B-mCherry) and RPE1-Venus-Parkin is also independent of Venus-Parkin as RPE1 cells stably expressing GFP-Mps1 are as competent as RPE1 (Venus-Parkin) to serve as the recipient cells (Supplementary Fig. S6f). This result suggests that the efficiency of this process is gene-specific in donor cells, but not in recipient cells. Future studies should pinpoint the genetic requirements for the robust transfer between RPE1 and MDA-MB-231 cells and whether other endogenous cellular genes can be transferred by this mechanism. Heterotypic or homotypic cell engulfment has been observed in certain tumor tissues^9^. It will be interesting to determine whether some of these interactions are reversible cell entrapment to fuel genetic heterogeneity and tumor evolution via HGT. The finding that MDA-MB-231 cells can dive into RPE1 cells via a transient entrapment process raises an interesting possibility that this could be a mechanism for tumor cells to breach the epithelial barriers and metastasize to distant organs. Future in vivo studies should be able to address the relevance of this mechanism in tumor metastasis.

## Supplementary information

Supplemental materials

Supplementary Information

Supplementary Table S1

Supplementary Movie 1

Supplementary Movie 2

Supplementary Movie 3
