# Supplementary material for: Cell type-specific intercellular gene transfer in mammalian cells via transient cell entrapment
Source: Cell Discov. 2022 Mar 1;8:20. doi: 10.1038/s41421-021-00359-x (PMC8885815; doi:10.1038/s41421-021-00359-x)
Supplement: Supplementary file 1 — Supplemental materials [file 41421_2021_359_MOESM1_ESM.docx]

**Supplemental materials:**

Cell-Type Specific Intercellular Gene Transfer in Mammalian Cells via Transient Cell Entrapment

Quanbin Xu^1^, Xiaojuan Zhang^1^, Gilson J Sanchez^2^, Adrian T Ramirez^2^, Xuedong Liu^1,*^

^1^ Department of Biochemistry, 3415 Colorado Ave, JSCBB, 596 UCB, University of Colorado, Boulder, CO 80303

^2^Department of MCD-Biology, University of Colorado, Boulder, CO 80309

^*^Corresponding author: Xuedong Liu

Tel: (303) 735-6161

E-mail: Xuedong.Liu@Colorado.Edu

Keywords: Cell entrapment, Gene transfer, Extracellular vesicles, ROCK1, ROCK2, Reverse transcription

**Supplementary information**

**Materials and Methods**

**Constructs, Stable cell line, and Cell culture**

The Parkin gene was inserted into retroviral expression vector pREX-Venus-DEST-IRES-Blasticidin and *mCherry*-tagged *H2B* was inserted into pREX-IRES-Hygromycin as described previously^1^. Cytosolic TagBFP was expressed using pCRISPRi/a-V2 (Addgene #84832). Two strategies were employed for gene knockdown: for *mCherry* mRNA knockdown, short hairpin (5’-CCGGGTGGGAGCGCGTGATGAACTTCTCGAGAAGTTCATCACGCGCTCCCACTTTTTG -3’) was cloned into pLKO.1-TRC (Addgene #10878); for *ROCK1/2* mRNA knockdown, the validated commercial siRNAs against ROCK1 (5’-GGUUAGGGCGAAAUGGUGUtt-3’) or ROCK2 (5’-GGAGAUUACCUUACGGAAAtt-3’) were purchased from ThermoFisher. The stable cell lines were constructed as described previously^1^. RPE-LAP-Mps1^as^ cell line was kindly provided by Drs. John Maciejowski and Prasad Jallepalli^2^. All cell lines were cultured in Dulbecco’s modified Eagle medium (DMEM) supplemented with 10% FBS, 2 mM glutamine, 100 U/mL penicillin, and 100 mg/mL streptomycin at 37ºC with 5% CO_2_ incubation.

**Cell synchronization**

Cells were synchronized at G1/S by single thymidine treatment for 24 hours. Cells arrested at G1/S were released into medium containing RO3306 for 19 hours and collected by shaking off to obtain mitotic cells.

**Flow cytometric analysis**

1 × 10^5^ RPE1-Venus-Parkin cells alone or along with MDA-MB-231-H2B-mCherry cells were seeded on 12-well plate and incubated for 2 days. These cycling populations were digested with trypsin/EDTA and then subjected to flow cytometric analysis after being resuspended with 0.5 mL DMEM.

**Karyotype analysis**

Cytogenetic analysis was performed on ten G-banded metaphase spreads of RPE1 and RPE1mut231 cell lines at passage 20. Karyotype analysis was performed by Karyologic, Inc (Research Triangle Park, NC).

**Retroviral integration site analysis**

Genomic DNA from MDA-MB-231 or RPE1 cells was isolated from subconfluent culture cells growing on 6 cm plate using a QIAamp DNA blood kit (QIAGEN). A Universal GenomeWalker^TM^ 2.0 kit (TakaRa, USA) was used to design and amplify the MoLV LTR junctional fragments from both cell lines prior to subcloning into a TA-cloning vector pCR2.1 (ThermoFisher) and sequencing.

**Live cell imaging**

For long-term imaging using an ImageXpress microscope, donor cells and recipient cells were seeded at a 1:1 ratio on glass-bottomed dishes (Mattek, Ashland, MA) in a 150 µL complete FluoroBrite DMEM and incubated/treated as shown in figure legends. All live microscopy was performed in an incubation chamber at 37°C, with 5% CO_2_ and for long-term imaging media was overlaid with mineral oil. Fluorescent images were acquired every 0.5 or 1 hour, and data analysis was performed with Image J software.

Confocal images were acquired on a Nikon A1R Confocal and TIRF using a 100X (NA 1.45) objective or Opera Phenix (PerkinElmer) using a 40X water objective. For immunofluorescence microscopy, cells were fixed with 4% paraformaldehyde. Immunofluorescence microscopy was performed as described previously^1^.

**Quantification and Statistical Analysis**

The donor to recipient cell ratio (R_d/r_) was calculated as the number of donor cells (Q3+Q4) divided by the number of recipient cells (Q1+Q2). The trendline and equation were generated using a built-in statistical tool in Microsoft Excel. To determine the effect of R_d/r_ ranges on gene transfer, the training data was generated as described in Methods and Materials and followed by one-way ANOVA and Post Hoc analysis (Table S1).

Unpaired t-tests were used to calculate *p*-values (depicted in Dataset S1) for each set of compared results. To determine the effect of donor to recipient cell ratio (R_d/r_) on gene transfer frequency, we used built-in statistical tool in Microsoft Excel to generate the trendline and equation. The training data were generated using the original data, original data with 90% SD, and original data with 110% SD to determine the effect of R_d/r_ ranges on gene transfer. These data were analyzed in GraphPad Prism using one-way ANOVA plus Post Hoc analysis. All other calculations, including average, SD, and P values, were performed using GraphPad Prism software (GraphPad Software, Inc.).

**References**

1. Xu, Q. *et al*. Regulation of kinetochore recruitment of two essential mitotic spindle checkpoint proteins by Mps1 phosphorylation. Mol Biol Cell 20, 10-20 (2009).

2. Maciejowski, J. *et al*. Mps1 directs the assembly of Cdc20 inhibitory complexes during interphase and mitosis to control M phase timing and spindle checkpoint signaling. J Cell Biol 190 (1): 89–100 (2010).

**Supplementary Figure legends**

**Figure S1**. Characterization of  the identity of cells with intercellular gene transfer. **a** Karyotype analysis of RPE1 and RPE1mut231(n=10). **b** The percentage of transduced cells at different time points of co-culturing. **c-d** Validation of RPE1mut231cells after isolation from co-cultured cells and subsequent passages (P2, P6, P10, and P20). RPE1-Venus-Parkin cells (R1) were co-cultured with MDA-MB-231-H2B-mCherry (M) for 0 and 48 hrs. Cells positive for both Venus-Parkin and H2B-mCherry signals were sorted and subjected to flow cytometry analysis (b) or Western blot analysis (c). **e**PCR amplification of *TP53* , *Venus*, and *mCherry* gene fragments from the genomic DNA of MDA-MB-231-H2B-mCherry (MDA), RPE1VP (RPE1-Venus-Parkin), or RPE1mut231. **f** Scheme of the integration sites of MoLV reporter transgene in RPE1mut231 and MDA-MB-231-H2B-mCherry. **g** The integration site of MoLV reporter transgene in MDA-MB-231-H2B-mCherry lands in Chromosome 11. One integration site of MoLV reporter transgene in RPE1mut231 was mapped on Chromosome 7. **h** Validation of the integration site in MDA-MB-231-H2B-mCherry (MDA) and RPE1mut231(Rm). The arrow shows the specific PCR product known as 3C2ndPCR (the integration site junctional fragment) exists only in RPE1mut231 cells; *, non-specific DNA amplification product; RPE, RPE1-Venus-Parkin.

**Figure S2**. Reverse transcription is required for the intercellular transfer of reporter gene. **a** Flow cytometric analysis of the intercellular gene transfer between RPE1-Venus-Parkin(RPE1VP) and MDA-MB-231-H2B-mCherry (MDA231-H2BmCherry) with mCherry mRNA depletion using stably expressed shRNA against luciferase (shLuc) or mCherry (shmCherry) .  **b** Statistical analysis of mCherry RNA knockdown efficiency in MDA-231-H2B-mCherry cells. **c** qPCR Quantification of mCherry mRNA levels in the MDA-MB-231-H2B-mCherry cells stably expressing shRNA against firefly luciferase (shLuc) or mCherry (shmCherry). Untreated RPE1mut231 was used as a control. **d** Statistical analysis of the effect of Stavudine on gene transfer between RPE1-Venus-Parkin and MDA-MB-231-H2B-mCherry (one-way ANOVA, p<0.0001). The R_d/r_ ratios range from 0.64 to 1.11, which have no statistical effect on gene transfer between the recipient and donor cells (Table S1, Post Hoc analysis, p=0.0618). **e** Effects of Stavudine on the reverse transcriptase activity in cell lysates from the donor and recipient cells. **f** Effect of Stavudine on the cell proliferation of RPE1-Venus-Parkin and MDA-MB-231-H2B-mCherry. Data are mean ± SD; statistical significance for (**b, c** and **e**) was assessed using the student’s *t-test* (***p<0.001, ****p<0.0001).

**Figure S3**. Direct cell-cell interaction is indispensable for intercellular gene transfer. **a** Semi-coculture assay. This assay allows two types of cells separated physically but still connected by the same medium. Briefly, 1% of percent of agarose in complete DMEM was casted into a 60mm dishes; holes were made using a 15ml conical tube after agarose solidified. RPE1-Venus-Parkin and MDA-MB-231-H2B-mCherry were then seeded into left and right holes respectively overnight. Then a bridge between two holes was created using a sterilized blade. **b** Statistical analysis of gene transfer between cells in semi-coculture assay.

**c, d**Schematic of experimental design for the effect of vesicles on gene transfer and the corresponding flow cytometric results. The media harvested from MDA-MB-231-H2BmCherry was centrifuged by 2000rpm to remove cell debris; the vesicles in the supernatant were further collected by 100,000x*g* centrifugation and then divided into two fractions; one was labeled with Ruby Red dye, and another was left untreated. These two samples were then co-cultured with RPE1-Venus-Parkin cells and followed by flow cytometric analysis. **e** Flow cytometry analysis and quantification of gene transfer between extracellular vesicles labeled or unlabeled and RPE1-Venus-Parkin cells. **f** Flow cytometric results of RPE1-Venus-Parkin or MDA-MB-231-H2B-mCherry culture separated by Transwell insert or co-cultured. Schematic of experimental design (upper panel) and a representative of flow cytometric result (lower panel). Data are mean ± SD; statistical significance for (**b** and **e**) was assessed using a student’s *t-test* (****p<0.0001).

**Figure S4**. Entrapment of MDA-MB-231 cells by RPE1 cells.  **a** Confocal images of coculture of RPE1-Venus-Parkin and MDA-MB-231-H2B-mCherry. These two types of cells were co-cultured for 12 hrs and then subjected to confocal analysis. **b** Confocal images of RPE1-Venus-Parkin and HeLa-H2B-mCherry which were co-cultured for 12 hrs before imaging. **c** Confocal images of coculture of RPE1-Venus-Parkin and MDA-MB-231-H2B-mCherry/CAAX-mCherry. **d** Live cell imaging of RPE1-Venus-Parkin and MDA-MB-231-H2B-mCherry/TagBFP. The nuclei of both cells were labeled with DRAQ5(pink).

**Figure S5.** ROCK kinases affect cell entrapment and intercellular gene transfer**. a** Flow cytometric analysis of gene transfer between RPE1-Venus-Parkin and MDA-MB-231-H2B-mCherry with siRNA against luciferase, *ROCK1*, *ROCK2*, or *ROCK1* plus *ROCK2*. **b** Western blots show the levels of ROCK1 or 2 in donor and recipient cells with siRNA targeting ROCK1(K1), ROCK2(K2), or both(K1&2). **c** Effect of ROCK kinase inhibitor Y27632 on gene transfer.  **d** Effect of ROCK kinases inhibition on cell-in-cell structure formation(100 cells/timepoint).  **e-h** Effect of the ROCK kinases knockdown or inhibition on the cell proliferation and motility of RPE1-Venus-Parkin(RPE1VP) and MDA-MB-231-H2BmCherry (MDA231-H2BmCh) cells. Data are mean ± SD; statistical significance for **h** was assessed using a one-way ANOVA analysis (****p<0.0001); ns, not significant.

**Figure S6 a** The effect of CDC42 GTPase inhibitor ML141 on gene transfer. RPE1 cells were cocultured with MDA-MB-231-H2B-mCherry (MDA) cells in the presence of ML141. ML141 shows a significant effect on gene transfer (p<0.0001). The inlet shows that the R_d/r_ ranges from 0.8 to 1.1, which doesn’t affect gene transfer significantly based on Post Hoc analysis (Table S1, p>0.05). **b** The list of genes screened for potential targets affecting gene transfer. The donor or recipient cells expressed validated shRNA or siRNA were co-cultured with the corresponding recipient or donor cells with siRNA/shRNA against luciferase. The gene transfer ratios were analyzed using flow cytometry analysis. The fold change of gene transfer ratio was given by the gene transfer ratio of the donor or recipient cells with specific shRNA/siRNA were divided by the ratio of donor cells and recipient cells with control siRNA. **c** Effect of cell cycle on gene transfer. The donor or recipient cells at the indicated cell cycle stage were co-cultured and the gene transfer ratio was measured using flow cytometry. Asyn, asynchronized cells.  **d** Flow cytometry analysis of RPE1-Venus-Parkin co-cultured with MDA-MB-231-H2B-mCherry(MDA231-H2BmCh), MDA-MB-231-αTubulin-mCherry (MDA-TubmCh), or HeLa- H2B-mCherry(HeLa- H2BmCh). **e** Cell type specificity of gene transfer. D. cells, Donor cells; R. cells, Recipient cells; n/a, not available. All donor cell lines stably express H2B-mCherry; SW480 and MCF7 consistently express YFP-Mps1.  **f** Flow cytometry analysis of coculture of RPE1-LAP-Mps1^AS^ cells(expressing fused GFP-Mps1 protein) and MDA-MB-231-H2B-mCherry (MDA231-H2BmCh) cells. Data are mean ± SD; statistical significance for **d** was assessed using the student’s *t-test* (****p<0.0001).

**Supplementary table 1**. Effect of donor to recipient cell ratio (R_d/r_) on gene transfer frequency.

**Supplementary Movie 1**: Live cell imaging of the entrapment of MDA-MB-231-H2B-mCherry by RPE1-Venus-Parkin.

**Supplementary Movie 2**: Live cell imaging of the entrapment of MDA-MB-231-H2B-mCherry/BFP by RPE1-Venus-Parkin.

**Supplementary Movie 3:** Live cell imaging of the interaction of RPE1-Venus-Parkin and HeLa-H2B-mCherry.
