# Supplementary figures and images for: Cell type-specific intercellular gene transfer in mammalian cells via transient cell entrapment
Source: Cell Discov. 2022 Mar 1;8:20. doi: 10.1038/s41421-021-00359-x (PMC8885815; doi:10.1038/s41421-021-00359-x)

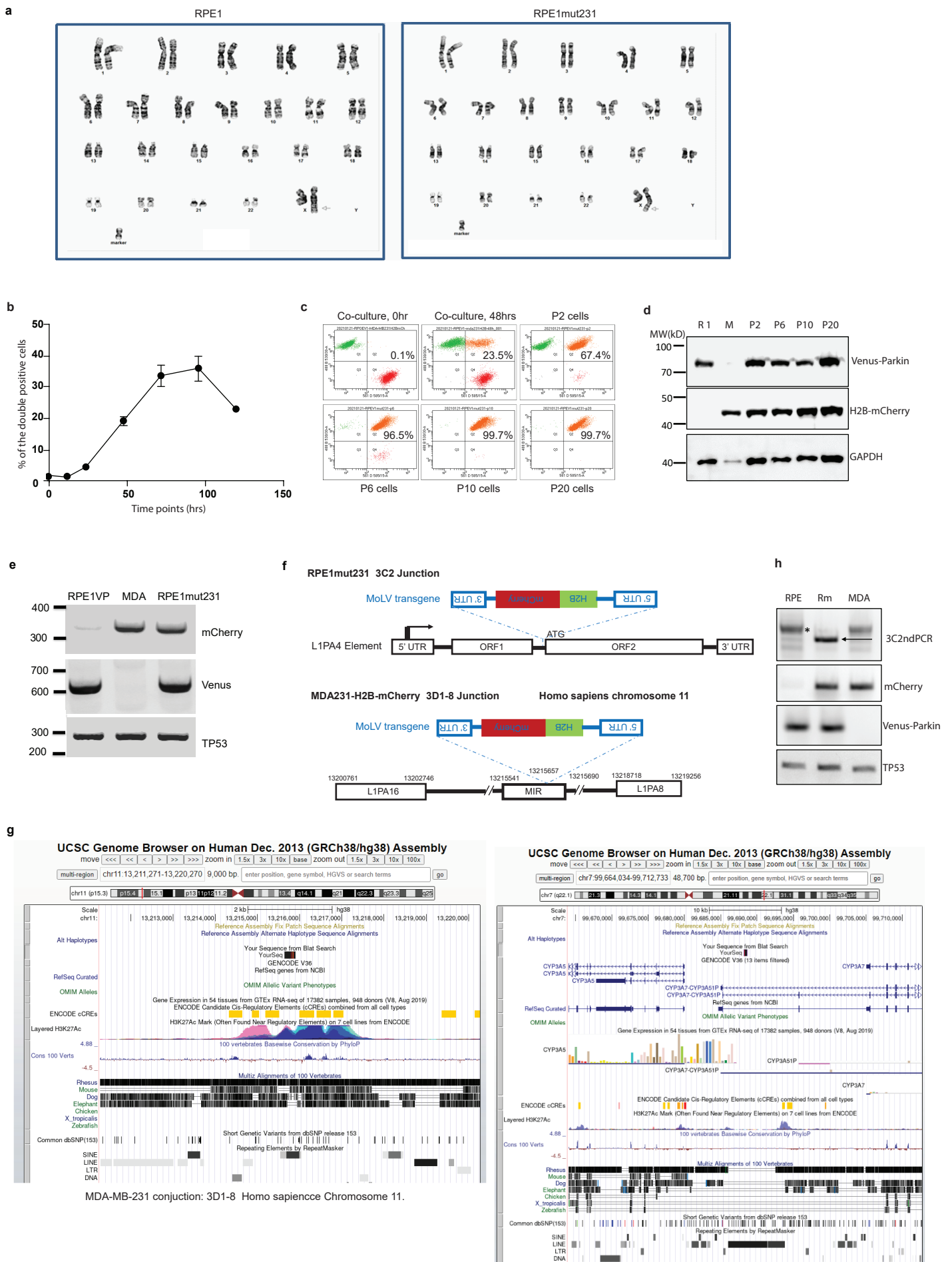

Figure S1

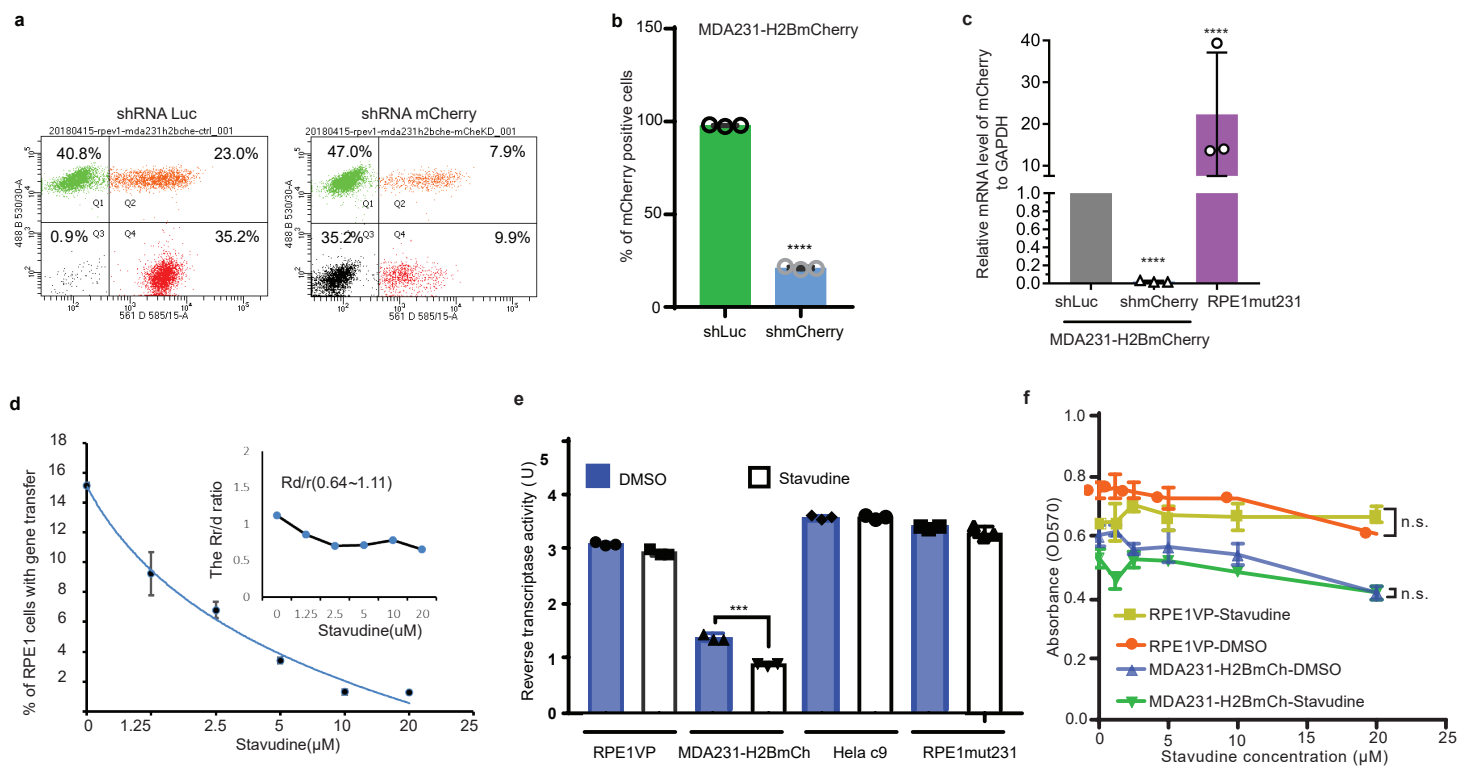

Figure S2

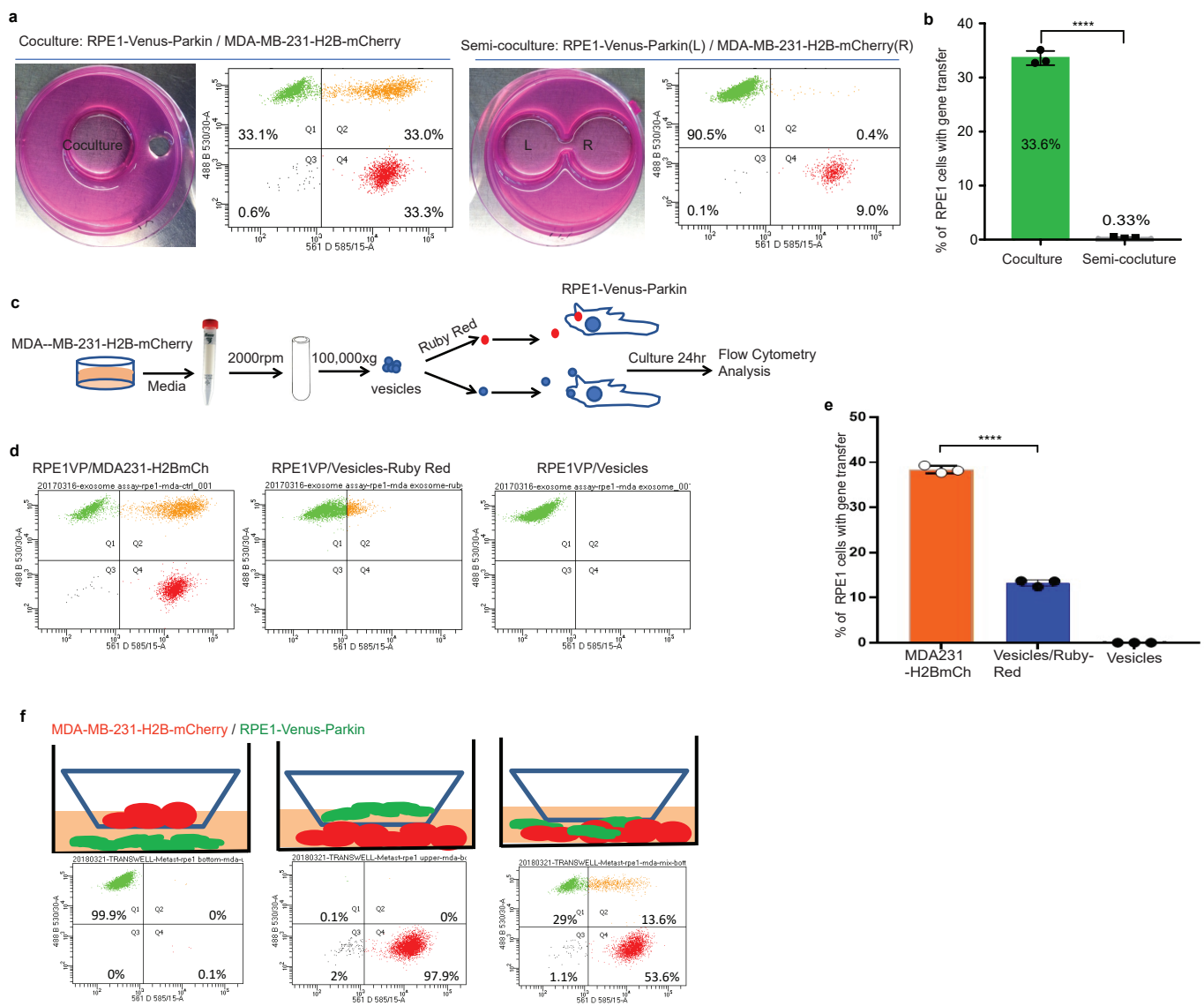

Figure S3

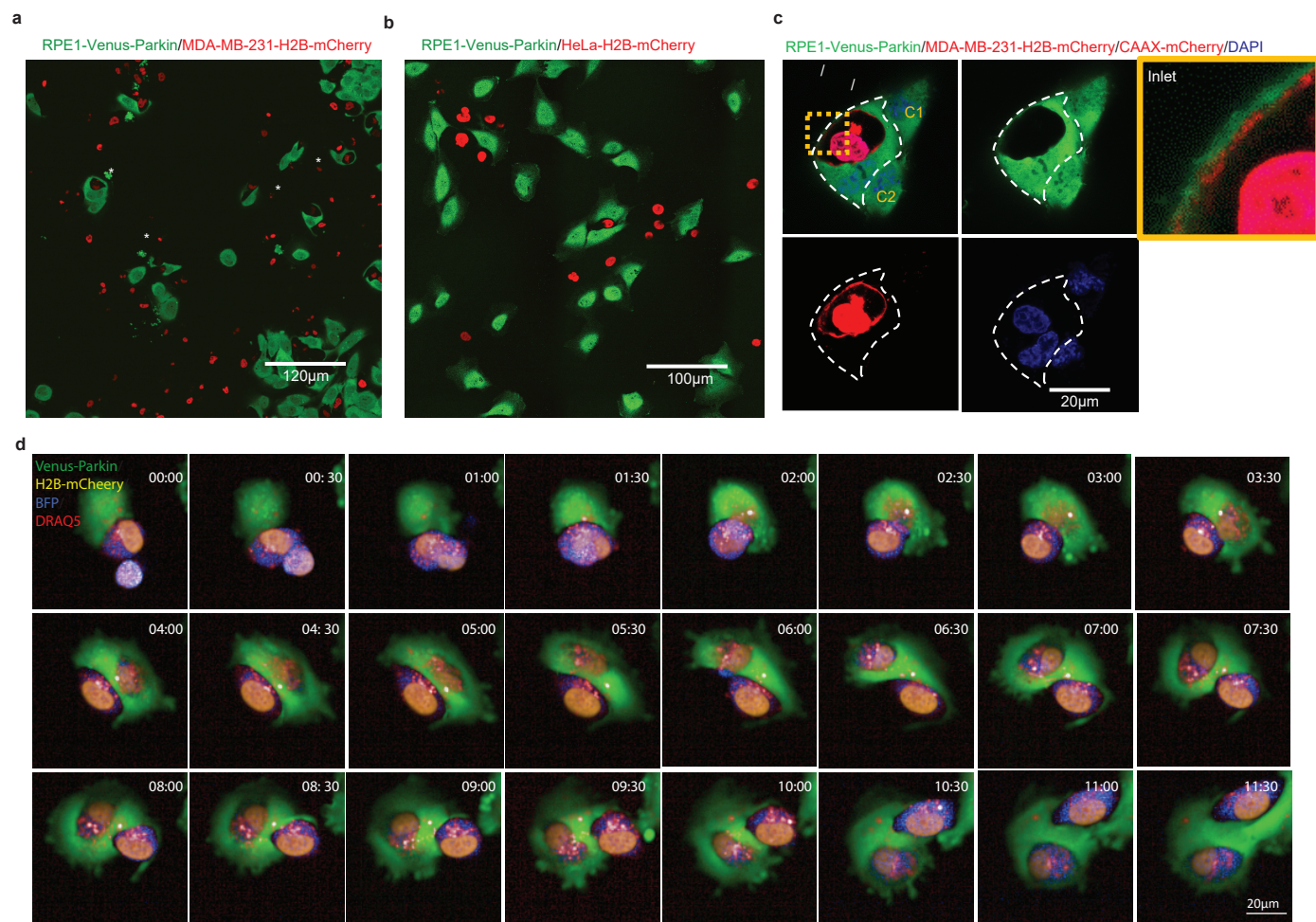

Figure S4

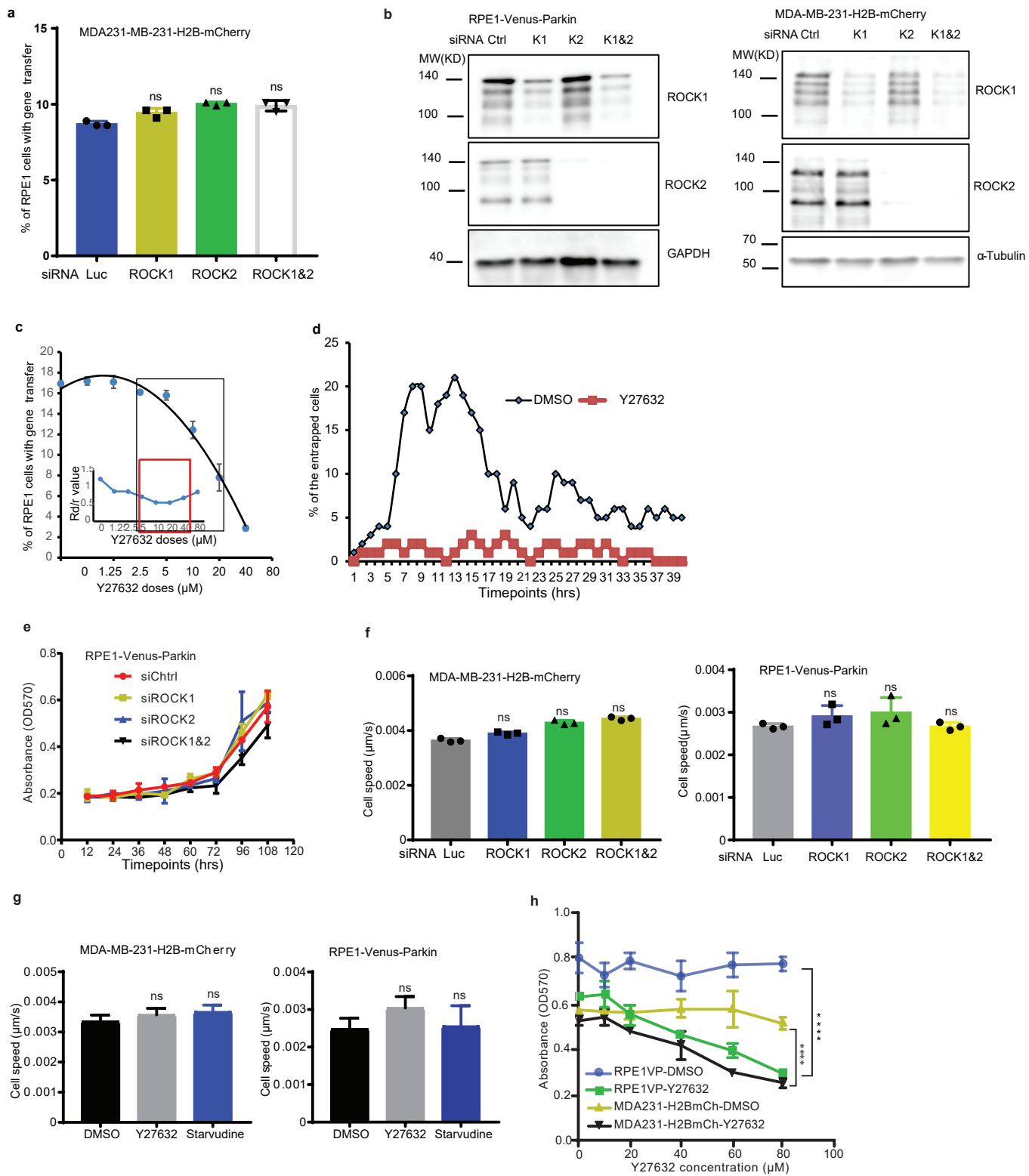

Figure S5

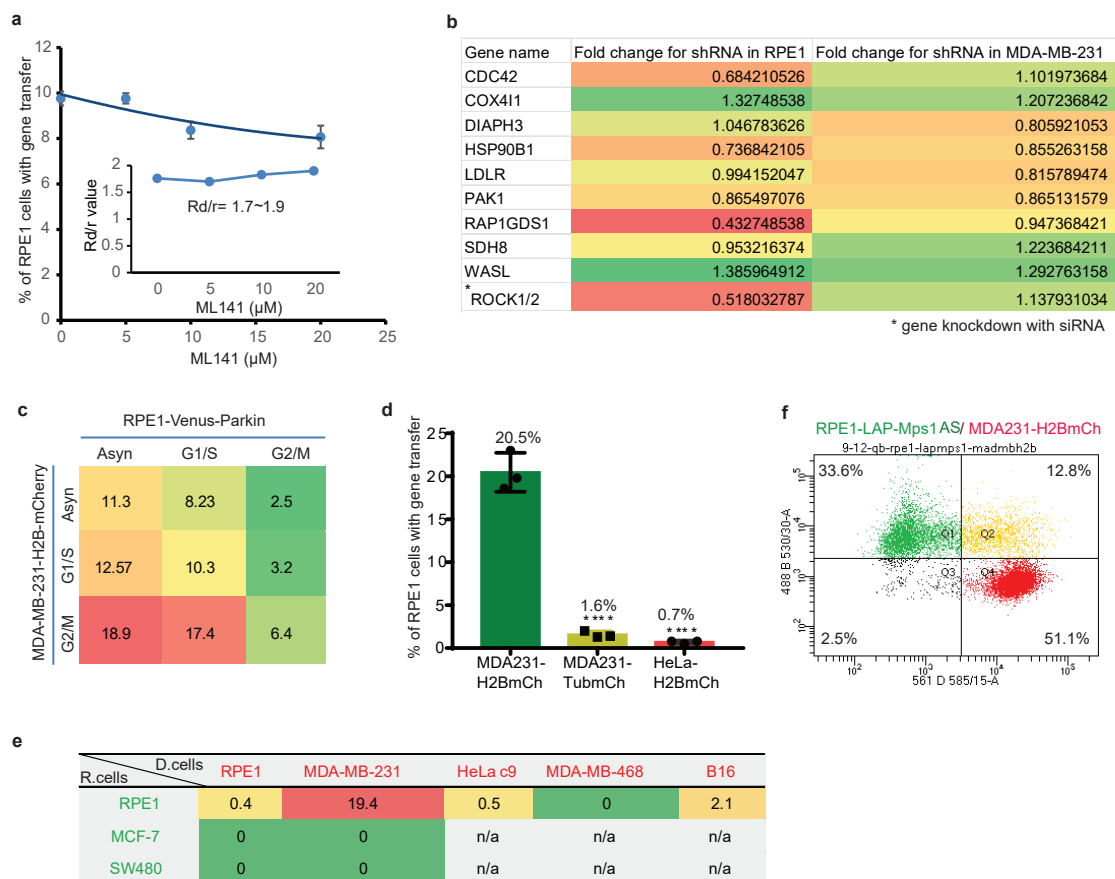

Figure S6

Supplement: Supplementary file 2 — Supplementary Information [file 41421_2021_359_MOESM2_ESM.pdf]
